# Health benefits of fermented olives, olive pomace and their polyphenols: a focus on the role of lactic acid bacteria

**DOI:** 10.3389/fnut.2024.1467724

**Published:** 2024-09-18

**Authors:** Federica Montagano, Francesca Dell’Orco, Roberta Prete, Aldo Corsetti

**Affiliations:** Department of Bioscience and Technology for Food, Agriculture and Environment, University of Teramo, Teramo, Italy

**Keywords:** lactic acid bacteria, fermented foods, table olives, olive pomace, polyphenols, prebiotic effects, health benefits

## Abstract

Fermented foods have regained popularity in Western diets for their health-promoting potential, mainly related to the role of lactic acid bacteria (LAB) during the fermentation process. Nowadays, there is an increasing demand for vegetable-based fermented foods, representing an environmentally sustainable options to overcome the limitations of lactose intolerance, vegetarian, or cholesterol-restricted diets. Among them, table olives and their co-products (i.e., olive pomace) represent important plant-origin matrices, whose exploitation is still limited. Olives are an important source of fiber and bioactive molecules such as phenolic compounds with recognized health-promoting effects. Based on that, this minireview offer a brief overview about the potential beneficial role of fermented table olives/olive pomace, with a particular focus on the role of LAB to obtain healthy and/or probiotic-enriched fermented foods.

## 1 Introduction

Fermented foods have been a part of human diets for centuries, with the knowledge and practices being passed down through generations (1) and still today, fermentation represents one of the most used biotechnological processes for the biotransformation of raw materials into nutritious, palatable and organoleptically satisfying products (2). Recently, fermented foods have been scientifically defined as “those foods or beverages made through controlled microbial growth and enzymatic conversions of major and minor food components” (3). Almost all populations have applied the fermentation process to vegetal (such as fruits, seeds, tubers, and other materials) and animal matrices (such as eggs, fish, meat, and milk) (4). Fermentation is most often accomplished with selected microorganisms, mainly lactic acid bacteria (LAB), that when added to the substrate as starter cultures produce lactic acid and, eventually, other organic acids such as acetic acid, besides ethanol and carbon dioxide, lowering the pH of food matrix which, in its turn, contributes to the safety and shelf-life extension of the final product (5). Specifically, LAB improve food stability via physical and biochemical changes in fermented foods, contributing also to their distinctive taste, flavor and texture (6, 7).

In addition, LAB metabolic activities are associated with production of many beneficial compounds such as polyols, exopolysaccharides and antimicrobial compounds (i.e., bacteriocins) (8). Indeed, many LAB strains have been studied for their health-promoting properties and recognized as potential probiotics based on well-established criteria (9, 10). LAB fermentation can improve the overall functionality of the food itself by the production of secondary bioactive metabolites (11), which can increase the nutritional value of the food and confer health benefits, showing to be able to modify gut barrier function and intestinal microbiota in a positive way to prevent and/or treat various metabolic and inflammatory diseases (12, 13). Moreover, during fermentation LAB can act synergistically with dietary fibers and/or non-fiber substances, such as polyphenols, in conferring also prebiotic effects, namely providing beneficial effect through a microbiota-mediated mechanism. Indeed, the current scientific definition developed by ISAPP in 2016 of a prebiotic as a “substrate that is selectively utilized by host microorganisms conferring a health benefit,” clarify the need to selectively elicit a limited group of microorganisms in the host rather than the entire microbial ecosystem (14). Based on that, it has been suggested that fermented foods should be included as part of national dietary recommendations for their outstanding role in human nutrition (3). Moreover, the wide variety of raw material-microbe combinations results in a multitude of different fermented foods and beverages and the expanding range of vegetable-based matrices has spurred new research into plant products as matrices for the production of fermented and probiotic products (5). In addition, plant-based fermented products can also be consumed by people with lactose intolerance and milk allergies (15).

Furthermore, there is a growing attention among the population for a more sustainable diet (i.e., vegetarianism, veganism, and flexitarianism) and for consuming low-cholesterol foods (16), and at the same time in line with the Mediterranean diet, associated with a reduced risk of developing chronic diseases and a longer life expectancy. Among the most popular plant-derived fermented products, table olives are one of the oldest fermented vegetable foods in the Mediterranean area, with a millenary tradition and a significant economic importance due to their high appreciation by consumers. Besides that, fermented table olives also represent an important fermented food included in the Mediterranean diet (17), whose guidelines suggest the daily consumption of 1–2 portions of olives, seeds and nuts as healthy snacks (18). The high nutritional value of table olives is mainly due to the presence of several compounds with biological and functional value (2, 19, 20), and the nutritional composition is mainly influenced by several factors, including agronomical factors, type of cultivar and the ripening stage of the drupes as well as the fermentation and the storage processes (17, 21, 22). In general, the main constituents of olive pulp are water (60–75%) and lipids (10–25%). More in details, lipids are the most important fraction in contributing to the nutritional value of table olives, and most representative are triglycerides, combined with a small amount of sterols, fatty and triterpenic alcohols (23). In particular, table olives’ lipids are characterized by a high level of unsaturated fatty acids, as monounsaturated fatty acids (MUFA) (i.e., oleic acid), polyunsaturated fatty acids (PUFA) as linoleic acid and saturated fats (i.e., palmitic acid, steric acid) at lower level (23).

Soluble reducing and non-reducing sugars, such as glucose, fructose, galactose, mannitol and sucrose, are present in the raw olive pulp (3–6%), but they result almost absent in fermented table olives, due to their solubilization during washing step and their transformation during fermentation and storage in brine. The overall high nutrition value of table olives is also due to the presence of all essential amino acids, even though total proteins are very low (21).

Moreover, table olives can be considered a reservoir of dietary fiber (2.5–5%), mainly pectin, hemicelluloses, cellulose and lignin, and in small amount table olives provide also vitamins such as *α*-tocopherol, *β*-carotene, vitamin B complex (i.e., thiamin, niacin, pantothenic acid, vitamin B6), minerals and microelements (24).

Besides nutrients table olives contain high amount of phenolic compounds, with demonstrated biological and functional value. They include flavonols (i.e., quercetin-3-rutinoside); flavones (luteolin and apigenin glucosides), anthocyanins (cyanidin-3-O-glucoside), phenolic acids (5-O-caffeoylquinic acid), phenolic alcohols (i.e., tyrosol, hydroxytyrosol), secoiridoids (i.e., oleuropein, demethyloleuropein) and a hydroxycinnamic acid derivative (verbascoside). Although their composition depends on the olive variety, during fermentation processes the phenolic composition of table olives changed. Depending on the method, phenols can diffuse in the brine, or they were hydrolyzed. In particular, hydroxytyrosol and tyrosol result the major phenols in the fermented product with a significant reduction of secoridoids (oleuropein, demethyloleuropein) and their aglycon derivatives (3,4-DHPEA-EDA) in the olive pulp mainly due to microbial enzymatic activities while the decrease of verbascoside, is related to its release in the brine after the process (22, 24, 25). Oleuropein, hydroxytyrosol and tyrosol are considered the most natural and powerful antioxidants and their biological role, related on the protective role against oxidative stress, lipoprotein metabolism, inflammation and blood pressure has been widely assessed (17, 24).

A recent pilot study based on the administration of 12 green table olives (cv. Nocellara del Belice) per day for 30 days in healthy adult subjects demonstrated an anti-inflammatory and antioxidant effects related to the content of polyphenols and monounsaturated oleic acid, suggesting a clear nutraceutical potential of this food (26).

In addition, the Mediterranean Diet promotes the centrality of the consumption of extra-virgin olive oil, whose production generates high amount of olive pomace as a main co-product, still very rich in monounsaturated fatty acids, and polyphenols and their derivatives (i.e., hydroxytyrosol, tyrosol, oleuropein, hydroxytyrosol glucoside, etc.) (27–29), whose role in disease prevention has been recently recognized with a claim by the European Food Safety Authority (EFSA) for their potential health benefits, mainly in reducing oxidative stress and inflammation (30), boosting the research interest on investigating their potential health benefits (27–29, 31–34).

Although the huge health potential of these food matrices, their application as vegetable-based functional foods is still limited. Although olive oil and its nutritional and non-nutritional components have been extensively investigated for their effects on human health, regarding fermented table olives there is a lack in *in vitro* studies (17) and only few studies have investigated the beneficial effects of table olives and olive pomace as fermented foods *in vivo* (31, 35) in either animal or human trials. With that in mind, this mini-review provides a brief overview about the potential beneficial role of fermented table olives/olive pomace as functional foods, with a particular focus on the role of LAB to obtain healthy and/or probiotic-enriched fermented foods.

## 2 Table olives and olive pomace as vehicle for probiotics

Diet may represent a vehicle of exogenous microorganisms with positive effects on human health, and probiotics and prebiotics are the most used strategies for this purpose. Generally, probiotics are bacteria isolated from human sources, mostly from the gastro-intestinal tract, and are defined as “live microorganisms which, when administered in adequate amounts, as part of a food or a supplement, confer a health benefit on the host” (2).

Several studies have showed how native strains isolated from table olives, mainly belonging to *Lpb. plantarum* and *Lacticaseibacillus casei* taxonomic groups also possess specific probiotic traits and showed adhesion and co-aggregation capabilities to form biofilms on the surface of the fruit (36, 37). This could be considered beneficial because their presence appeared to effectively inhibit the adhesion of undesirable microorganisms during storage (38).

Scientific evidence has been reported on the genetic features of LAB strain adhesion on olive surfaces.

Different studies revealed that genes *eno*A1, *gpi* and *oba*C were necessary in *Lpb. pentosus* to form an organized biofilm on the olive skin during fermentation (39), as well as the presence of four moonlighting proteins over-produced in *Lpb. pentosus* strains isolated from green table olives (38).

Therefore, the above mentioned properties turn table olives into valid carriers of potential probiotics (2, 38, 40–48). In fact, over the last 10 years, an increasingly growing number of experimental studies have emerged to evaluate the suitability of LAB probiotic strains to be inoculated during the fermentation (Table 1). Montoro et al. (49) have investigated the probiotic potential of 31 different *Lpb. pentosus* strains, which exhibited good growth capacity and survival under gastro-intestinal conditions, adherence to intestinal and vaginal cells, auto-aggregation and co-aggregation due to the presence of key protein involved in mucus adhesion. In addition, these microorganisms showed the capacity to improve lactose digestibility (38, 49). Other study revealed high cell viability on table olive surfaces of microencapsulated *Lpb. pentosus* strain that also exhibits antioxidative ability, antibiotic resistance and survivability after simulated digestion (50). Nikfarjam et al. (51) have demonstrated that the using of *Lpb. plantarum* PGNM8 in fermented olive as a starter with probiotic potential improved remarkably organoleptic properties of the product as well as had suitable survival in fermented olive containing NaCl (6%) after 75 days (10^6^ CFU/mL) (51).

**Table 1:** Experimental studies for the development of functional/probiotic olives.

Potential probiotics	Outcomes and bioactivity	References
**Table olives fermentation**		
*Lpb. plantarum* PGNM8	Faster acidification and reduced possibility of spoilage Improved organoleptic properties of fermented olive Survivability in fermented olive containing NaCl (6%) after 75 days (10^6^ CFU/mL)	Nikfarjam et al. (51)
*Lpb. plantarum* Lp 15-Lp 20 - Lp 28-Lp 40-Lp 48	Reduction of the fermentation time Decrease the risk of *Enterobacteriaceae* spoilage Increase in hydroxytyrosol and tyrosol Inactivation of *E. coli* O157 EDL-932 and *L. monocytogenes* ScottA	Tataridou and Kotzekidou (52)
*Lpb. pentosus* B281, *Lpb. plantarum* B282	Faster acidification and survive at high numbers (>6 Log UFC/g) at the end of the fermentation and during the storage Sensory acceptability of fermented olives	Argyri et al. (41), Blana et al. (53), Argyri et al. (81), Blana et al. (82)
*Lpb. pentosus* CF2-10N, CF1-6, AP2-16N, CF1-43N	Good growth capacity and survival under gastro-intestinal conditions Auto-aggregation and co-aggregation due to the presence of key protein involved in mucus adhesion Adherence to intestinal and vaginal cells Improving lactose digestibility	Pérez Montoro et al. (38), Montoro et al. (49)
*Lpb. pentosus* i106	Benefits as a supplementary additive or adjunct culture	Coimbra-Gomes et al. (83, 84)
*Lcb. paracasei* IMPC2.1	Colonized the olive surface dominating the natural LAB population Survived on the matrix with a load ≥7 log CFU/g Survived simulated gastro-intestinal digestion	De Bellis et al. (54)
*Lpb. pentosus* NRRL B-227	High cell viability of microencapsulated *Lpb. pentosus* on table olive surfaces (8 log CFU/g) after 72 weeks of storage Antibiotic, antioxidant, and digestion fluids resistant properties	Elvan et al. (50)
*Lpb. pentosus* TOMC-LAB2	Survived during shelf-life at room temperature Sensorial profile acceptability Strain predominance influenced by olive maturation and the type of inoculum	Rodríguez-Gómez et al. (85, 86), López-López et al. (87)
*Lpb. plantarum* UT2.1 *- Lcb. paracasei* N24 *- Lpb. pentosus* TH969	Faster acidification High biodiversity that positively correlates with ester compounds which give fruity and floral aromas Preventing *Enterobacteriaceae* growth at the end of fermentation	Randazzo et al. (88, 89)
*Lcb. paracasei* N24	Higher acidification and debittering Strong oleuropeinolytic activity at low salt concentration Enhanced VOCs and sensory profile High viability in the final product	De Angelis et al. (57), Pino et al. (90)
*Lcb. rhamnosus* H25*, Lcb. rhamnosus* GG	Impact on VOCs and sensory characteristics Survival during fermentation of table olives	Randazzo et al. (56)
*Lpb. pentosus* LPG1*, Lpb. pentosus* Lp13*, Lpb. plantarum* Lpl15*, W. anomalus* Y12	Rapid acidification, sugar consumption, LAB growth Enhanced biofilm formation with the production of Autoinducer-2 (AI-2) as signaling molecule	Benítez-Cabello et al. (46, 91)
*Commercial Lpb. plantarum-W. anomalus* DiSSPA73 (SY)*; commercial Lpb. plantarum-W. anomalus* DiSSPA73*-Lpb. plantarum* DiSSPA1A7*-Lpb. pentosus* DiSSPA7 (SYL)	Sweeter taste perception and the highest sensory appreciation for SYL Increase in some phenolic and volatile compounds for SY and SYL Increase in carotenoids and FAA	De Angelis et al. (57), Cosmai et al. (58)
*Leu. mesenteroides* K T5-1*, S. cerevisiae* KI 30-16*, Lpb. plantarum* A135-5*, Debaryomyces hansenii* A15-44	Increased hydroxytyrosol and tyrosol with sequential inoculation (first yeast, then LAB) Enhanced antioxidant content	Chytiri et al. (92)
**Olive pomace fermentation**		
*Lpb. plantarum, W. anomalus, C. boidinii*	Increase % alcohol content produced by yeasts Increase % esters content produced by *Lpb. plantarum* Increase inhibition of COX-2 and antioxidant activity	Tarantini et al. (59)
*Leu. mesenteroides* KT 5-1, *Lpb. plantarum* BC T3-35, TB 11-32, C 180-11, *Saccharomyces cerevisiae* LI 180-7, KI 30-16, *D. hansenii* A15-44	Increase total polyphenolic compounds produced in spontaneous and controlled fermentation also after pasteurization Increase antioxidant activity Excellent source of health promoting compounds (i.e., hydroxytyrosol and tyrosol)	Tarantini et al. (59), Tufariello et al. (60), D’Antuono et al. (61)

In their study, Tataridou and Kotzekidou (52) have used oleuropeinolytic strains of the *Lpb. plantarum* group as both the debittering agent of black and green olives. The results show the ability of these strains to increase biophenols, especially hydroxytyrosol and tyrosol, and inactivate the pathogens *Escherichia coli* O157 EDL-932 and *Listeria monocytogenes* ScottA (52).

Other researchers evaluated the performance of *Lpb. plantarum* and *Lpb. pentosus* strains co-inoculated in olive fermentations. Specifically, the probiotic strains, *Lpb. pentosus* B281 and *Lpb. plantarum* B282, could be considered also promising starter cultures to produce a high added value final product with improved sensory profile and probiotic potential. Among the two strains, *Lpb. pentosus* B281 was able to survive better in the different salt levels employed in fermentation and to colonize the olive surface at higher concentration compared to *Lpb. plantarum* B282, dominating even in the case of mixed inoculum (41, 53).

Among others, *Lacticaseibacillus paracasei* strains are assessed for their potential probiotic role in olive fermentations, also because this species has a close taxonomical relationship with *Lcb. casei*, involved in the natural fermentation of table olives (54). Indeed, De Bellis et al. (54) have investigated the dynamics of microbial populations adhering on the surface of debittered green olives cultivar cv. Bella di Cerignola combined with the probiotic strain *Lcb. paracasei* IMPC2.1 during the fermentation. The results illustrated a successfully colonization of the olive surface dominating the natural LAB population, establishing an accelerate fermentation process and reducing the survival period of potential spoilage microorganisms.

Moreover, related works concern the addition of *Lcp. paracasei* N24 strain in Sicilian table olives and its persistence after fermentation. This strain shows high acidification and debittering rate, strong oleuropeinolytic activity at low salt concentration and increased volatile organic compounds (VOCs), contributing to more pleasant flavors (54, 55).

Among the different species that predominate in table olives, *Lcb. rhamnosus* is not commonly found, but in their study Randazzo et al. (56) demonstrated that two probiotic *Lcb. rhamnosus* strains were able to survive during fermentation, even if their main ecological niche is dairy products. The presence of *Lcb. rhamnosus* at the end of the process is therefore attributed to the artificial addition of probiotics (56).

Recently, it has also been suggested the use of yeasts in combination with LAB to enhance the organoleptic quality and shelf-life of table olives (57, 58).

In their experimental study, De Angelis et al. (57) used different combination of potential probiotic LAB and yeasts with positive effects on the taste perception and sensory profile of table olives and olive pomace. The results highlight an increase in some volatile organic compounds, in carotenoids and free amino acids (FAA) (57, 58). Other studies applied a combined inoculum of LAB and yeasts to ferment table olives with beneficial impact on health-promoting compounds, antioxidant activity and total polyphenolic content (59–61).

Concerning by-products of olive oil process, Foti et al. (62) fermented the pâté olive cake with different microbial strains (*Lpb. plantarum, Wickerhamomyces anomalus and Candida boidinii*) obtaining an improvement of the VOCs profiles and the biological activity, as the antioxidant and anti-inflammatory potential. In particular, *Lpb. plantarum* positively affect the ester concentration whereas the yeasts mostly affect the alcohols (62).

Thus, all this experimental evidence suggests that the application of microorganisms characterized by probiotic properties could significantly improve the functional characteristics of table olives and their derivatives, conferring greater health benefits, opening a novel scenario on the development of functional/probiotic table olives and related matrices as healthy vegetable-based fermented foods.

## 3 Olive’s health benefits and LAB

The use of plant-based matrices and LAB in the production of fermented foods is an ancient tradition that is now being supported by scientific understanding and technological advancement (63).

Some plant-based matrices present undesirable properties that LAB species have proven to ameliorate by their fermenting action. In particular, olives and their co-products (i.e., olive pomace) contain high amount of bitter secoiridoids that must be hydrolyzed before consumption in order to reduce and/or remove the olives bitterness and, in turn achieve acceptable palatability. Following IOC standard procedures (64) olive debittering can be carried out through chemical hydrolysis by adding alkalin solution (2.5–3% w/v) followed by spontaneous fermentation (3–7 months) driven by LAB (Spanish-style method) (65, 66) or in the Greek-style method, the bitterness is removed by the enzymatic activities of the olives microbiota combined with the diffusion of polyphenols into a saline brine (6–10% w/v) in which olives are directly soaked, during a long spontaneous fermentation (8–12 months) driven by LAB and yeast (65).

More recently, the biotransformation using selected LAB as starter cultures with oleuropenolytic activity to drive olives and olive pomace fermentation has emerged as a valid and sustainable process, being highly recommended for table olives fermentation (2). The careful selection of microbial strains with inherent desirable characteristics has a critical impact on a predictable and reproducible improvement of the different quality and nutritional attributes of fermented products (27, 28). In fact, an appropriate inoculum (usually 6–7 log CFU/ml) reduces the phenomena of spoilage by deteriorating microorganisms, inhibits the growth of pathogenic microbes and contributes to obtain a controlled and standardized process, improving the sensorial and hygienic quality of the final product and significantly reducing fermentation times (2). LAB starter cultures are usually represented by a single strain or by a mixture of strains in a limited number selected based on their technological aptitude and their suitability to be cultivated, and subsequently used as an inoculum to accelerate and improve the fermentation process (17). The use of selected starter cultures, significantly improve the fermentation process, allowing to overcome unexpected outcomes due to the variability of spontaneous fermentation that occurs in both Spanish-style and Greek-style method (2, 22, 65).

Among LAB, *Lactiplantibacillus (Lpb.) pentosus* and *Lpb. plantarum* are the most characterized and used species as starter cultures, as a single inoculum or in combination with other bacterial or yeast species to drive the biological debittering process of olives (2). The use of these species is mainly due to their enzymatic (*β*-glucosidase and esterase) activities (67), able to degrade oleouropein and, in turn, release elenolic acid and hydroxytyrosol (65), recognized as beneficial compound against oxidative stress and inflammation (30), to their high survival capacity in the fermentation environment (low pH and high concentrations of salts), which allows, thanks to a greater rapidity of growth, to quickly dominate the endogenous microbiota present in the fermentation brines. Furthermore, these species are equipped with additional specific enzymatic activities to produce volatile compounds which contribute to the development of the sensorial characteristics of the product (68). Moreover, the LAB-mediated fermentation process can enhance the nutritional and functional value of the raw foods, by the production of a high portion of other beneficial substances such as phenolic acids, bioactive peptides and short-chain fatty acids (SCFAs), among others (69).

Several studies reported that the beneficial properties (i.e., antioxidant and anti-inflammatory activity) in plant-based matrices can be increased by using *Lpb. plantarum,* alone or in combination with other LAB, in the fermentation process (32–35, 63, 65, 67, 69, 70).

Moreover, the application of bacteria characterized by probiotic properties could significantly improve the functional characteristics of table olives and their derivatives, conferring greater health benefits.

In our latest research, we have shown that the combined oral administration of a diet enriched with biologically debittered olive pomace and a specific probiotic strain (*Lpb. plantarum* IMC513) has a synergistic impact with anti-inflammatory and antifibrotic effects in a DSS-induced chronic colitis mice model (31). In addition, biologically debittered olive patè enriched with *Lpb. plantarum* with probiotics features showed to positively modulate intestinal human microbiota with a clear prebiotic effect (by increasing the abudance of *Lactobacillales, Bifidobacteriaceae, Akkermansia municiphila* and *Faecalibacterium prausnitzii*) and a positive correlation with the increased production of beneficial metabolites (i.e., SCFAs) in an *ex-vivo* gut model, confirming olive patè to be a good matrix for deliver beneficial microbes and, in turn, for the development of innovative fermented functional foods (32).

A similar *ex-vivo* fecal fermentation model has been also used by Ribeiro et al. (71) showing gastro-intestinal health benefits and prebiotic effects of olive pomace powders related to the stimulation of SCFAs production by gut microbiota as well as to asses the prebiotc role of olive pomace-enriched bread by Nissen et al. (72).

Similar prebiotic effect has been confirmed also *in vivo* in spontaneously hypertensive rats, after 7 weeks of daily intake of Arbequina table olives subjected to natural fermentation (73). The consumption of table olives promoted selectively the growth of intestinal beneficial microbes, such as *Lactobacillus* spp., *Bifidobacterium* spp. and *Akkermansia muciniphila* among others, with a related reduction in plasmatic concentrations of malondialdehyde and angiotensin II, demonstrating a clear antihypertensive activity (73), mainly due to the high amount of bioactive compounds, such as polyphenols delivered by table olives.

Olive polyphenols have been used to enrich yogurt, demonstrating a clear impact on body weight, body mass index, blood pressure, low density lipoprotein (LDL) cholesterol as well as a prebiotic effect by selectively increasing LAB population after 2 weeks of daily intake in a randomized, double-blind, placebo-controlled, crossover trial (74). This study indicates that 50 mg/day of olive polyphenols can help in decreasing LDL cholesterol and lipid peroxidation in healthy subjects.

Previously, a long term (12 months) administration of an olive polyphenol extract (Bonolive^®^) showed to improve lipid profile by decreasing total and LDL cholesterol in a double blind, randomized trial involving postmenopausal women (75). Hydroxytyrosol from olives fruit has been investigated for its potential cardioprotective effects in hypercholesterolemic (76) and hypertensive patients (77) in two pilot studies, confirming the positive effects of olives bioactive compounds in reducing LDL and preventing oxidation and inflammation, suggesting a promising use in the clinical management of hypercholesterolemic, hypertensive and metabolic disease.

More recently, the potential therapeutic role of these bioactive compounds, due to their antioxidant and anti-inflammatory properties, is emerging also as a potential strategy in the management of irritable bowel syndrome (IBD), for which successful and resolutive therapies are still missing (78). Up to now, experimental evidence are limited to intestinal cells and IBD-animal models that have indicated promising beneficial effects of olive polyphenols, (i.e., hydroxytyrosl, oleuropein) in decreasing pro-inflammatory cytokines (i.e., IL-1β, IL-6, IL-8, IL-17, TNF-*α*) and triggering NF-κB signaling and p38 MAPK pathway (68, 79, 80).

Based on that, the combination of bioactive compounds and beneficial microbes delivered by table olives and olive pomace can provide to the olive industry a new image of table olives and olive cream as health-promoting fermented foods with high nutraceutical value, with promising therapeutical application even though clinical evidence remain still limited (Table 2), and more well-designed randomized clinical trials are needed to confirm these beneficial effects.

**Table 2:** *Ex-vivo* and clinical studies investigating the health benefits of olive-derived products.

Study	Olive products	Timeline	Sample type or subjects	Outcomes	References
***Ex-vivo***					
	Fermented olive pomace	24 h	Feces from healthy donors	Prebiotic effect by increasing the abundance of beneficial bacterial groups, such as *Bifidobacteriaceae, Lactobacillales, Akkermansia muciniphila* and *Faecalibacterium prausnitzii* Increased production of beneficial SFCAs Decrease of detrimental VOCs related to proteolytic fermentations (i.e., indole, skatole)	Nissen et al. (32)
	Olive pomace	24 h	Feces from healthy donors	Prebiotic effect due to the SCFAs production by gut microbiota Bioactive phenolic compounds with potential antioxidant activity and antiadhesion ability against food pathogens	Ribeiro et al. (71)
	Olive pomace-enriched bread	24 h	Feces from healthy donors	Prebiotic effect by increasing the abundance of beneficial bacterial groups, such as *Bifidobacteriaceae* and *Lactobacillales* Increased production of SCFAs and reduction in abundance of harmful Branched-Chain Fatty Acids (BCFAs), indole, and skatole	Nissen et al. (72)
**Clinical**					
	Green table olives	30 days	Healthy subjects	Significant modulation of malondialdehyde with antioxidant effect Significantly decreased level of interleukin-6 Reduction of fat mass with an increase of muscle mass No statistically significant differences in the abundance of Lactobacilli	Accardi et al. (26)
	Olive fruit polyphenol-enriched yogurt	2 weeks	Healthy subjects	Significantly reduction of body weight, body mass index, hip circumference and systolic blood pressure Decreased levels of LDL cholesterol and thiobarbituric acid reactive substances increased abudance of LAB in yogurt	Georgakouli et al. (74)
	Olive polyphenol extract	12 months	Post-menopausal women	Increased levels of the pro-osteoblastic marker osteocalcin Decrease in total-and LDL-cholesterol	Filip et al. (75)
	Olive fruit extract	8 weeks	Hypercholesterolemic patients	Protection of LDL cholesterol from oxidation Prevention of the inflammatory status	Fonollá et al. (76)
	Olive fruit extract	2 months	Hypertensive and diabetic patients	Reduction of systolic and diastolic blood pressures Improved markers of hypertension and metabolic syndrome	Hermans et al. (77)

## 4 Conclusion/future perspective

Consumers are increasingly interested in a healthy diet, mainly by decreasing their dietary intake of high-fat and animal-based food products, due to health, sustainability and ethical concerns. Plant-based matrices are in line with those consumers’ needs, and among them, olives represent suitable matrices to produce healthy innovative and environmentally sustainable fermented foods. Table olives represent an important fermented food included in the Mediterranean diet as a source of fiber and bioactive molecules such as phenolic compounds with recognized health-promoting effects.

The transformation of these products through fermentation and the exploitation of the potential combined effects of olive bioactive compounds and olive-associated LAB would lead (1) to overcome limitations on their consumption, (2) to open new market trends by meeting the needs of lactose free, vegan-vegetarian or low-cholesterol diets, (3) to improve adherence to the Mediterranean Diet, despite the constraints of modern society, and (4) to suggest a potential therapeutical application in dietary intervention (i.e., hypercholesterolemia, IBD).
